# Cytomegalovirus colitis in an immunocompromised patient presenting with massive lower gastrointestinal bleeding

**DOI:** 10.1016/j.idcr.2022.e01500

**Published:** 2022-04-09

**Authors:** Malisa Surapatpichai, Sasathorn Taeudomkul, Chutima Jiragawasan, Thiyaphat Laohawetwanit

**Affiliations:** a Chulabhorn International College of Medicine, Thammasat University, Pathumthani, Thailand; b Division of Pathology, Thammasat University Hospital, Pathumthani, Thailand

**Keywords:** CMV colitis, Morphology, Histopathology

## Abstract

A 48-year-old man with HIV infection (CD4 count = 84 cells/μL) experienced hematemesis and hematochezia. Colonoscopy revealed massive bleeding in the colon, in which the source of the bleeding could not be identified. A total colectomy was performed. A large superficial ulcer at the rectosigmoid colon was observed. Histologically, abundant cytomegalovirus (CMV)-infected cells were noted. The pathological diagnosis was CMV colitis.

A 48-year-old man with HIV infection (CD4 count = 84 cells/μL) presented with hematemesis and hematochezia. An emergency endoscopy was performed. Esophagogastroduodenoscopy showed a clean base ulcer in the stomach. Colonoscopy revealed massive bleeding in the colon, in which the source of the bleeding could not be identified. CT of the whole abdomen displayed bowel wall thickening along the sigmoid colon and rectum. Later, the patient developed hypovolemic shock. A total colectomy was performed.

Grossly, a large ulcer (15×7 cm) at the rectosigmoid colon was observed (Fig. 1). This superficial lesion involved the mucosal and submucosal layers and spared the muscular layer of the rectosigmoid colon (Fig. 2, Fig. 3). Histologically, the superficial portion of the ulcer was covered by fibrinopurulent exudate. The deeper part contained granulation tissue, characterized by an admixture of mononuclear inflammatory cells and capillaries (Fig. 4). Abundant cytomegalovirus (CMV)-infected cells were observed. Most of them were infected mesenchymal and endothelial cells showing large ovoid nuclei with basophilic intranuclear inclusions (Cowdry bodies) surrounded by a clear halo (Fig. 5, Fig. 6). Intracytoplasmic inclusions were noted (Fig. 7). CMV immunostain highlighted these infected cells (Fig. 8). A CMV PCR was not done on the blood. The patient was discharged and lost to follow-up.

**Fig. 1:**
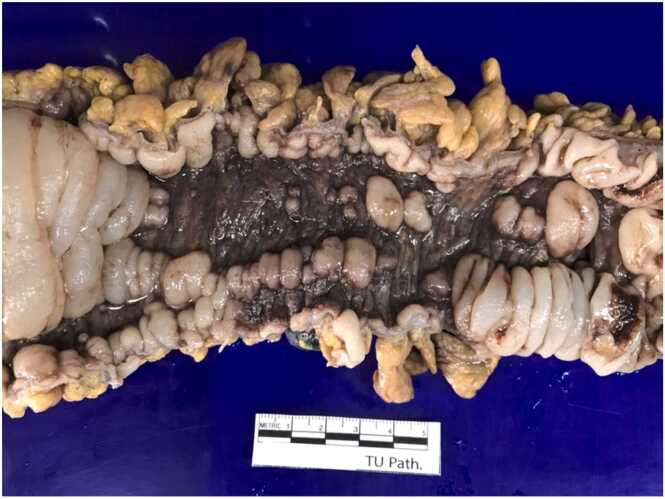
Ulcer at the rectosigmoid colon. Note a large ulcer (15 ×7 cm) at the rectosigmoid colon.

**Fig. 2:**
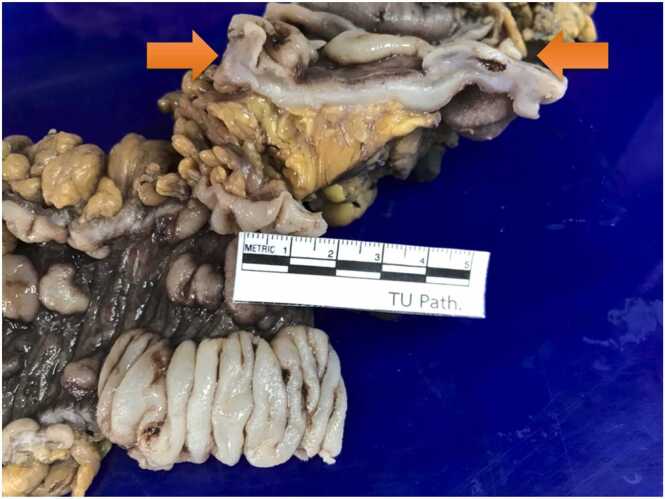
Cut surface of the ulcer. Grossly, the superficial ulcer involves mucosal and submucosal layers (arrows). No fissuring ulcer is noted.

**Fig. 3:**
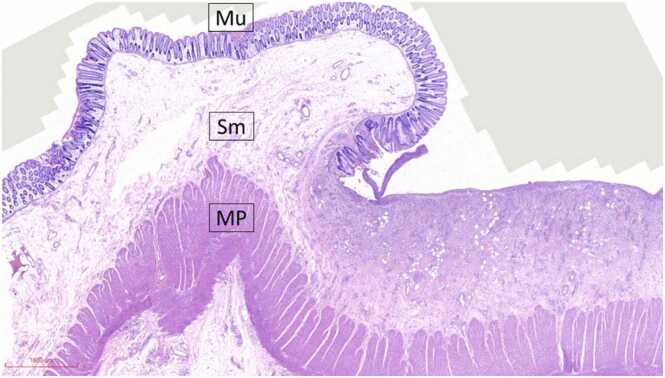
Photomicrograph of the transitional area. Histologically, the ulcer involves only the mucosal (Mu) and submucosal (Sm) layers. The muscularis propria (MP) is uninvolved.

**Fig. 4:**
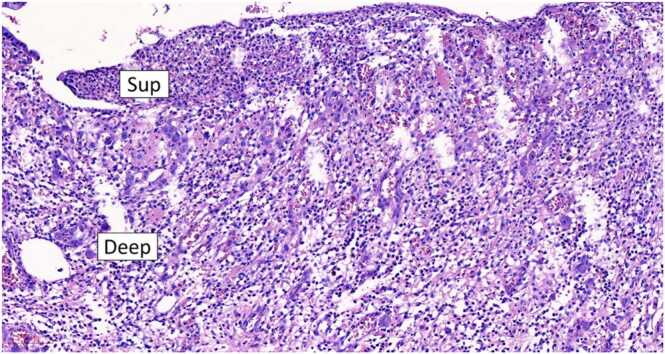
Photomicrograph of the ulcer. The superficial portion (Sup) of the ulcer is covered by fibrinopurulent exudate. The deeper part (Deep) contains granulation tissue, characterized by an admixture of mononuclear inflammatory cells and capillaries.

**Fig. 5:**
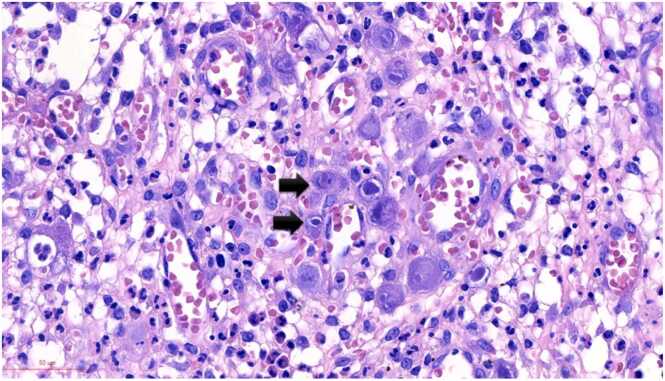
CMV infected mesenchymal cells. These CMV infected cells have large ovoid nuclei with basophilic intranuclear inclusions (Cowdry bodies) surrounded by a clear halo (arrows).

**Fig. 6:**
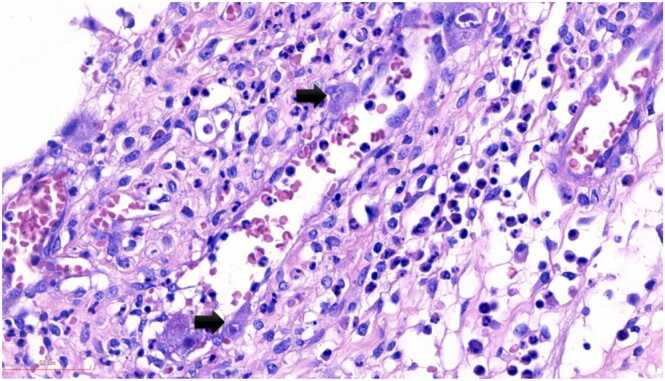
Endothelial cells infected with CMV. Endothelial cells, lining a capillary, are infected with CMV (arrows).

**Fig. 7:**
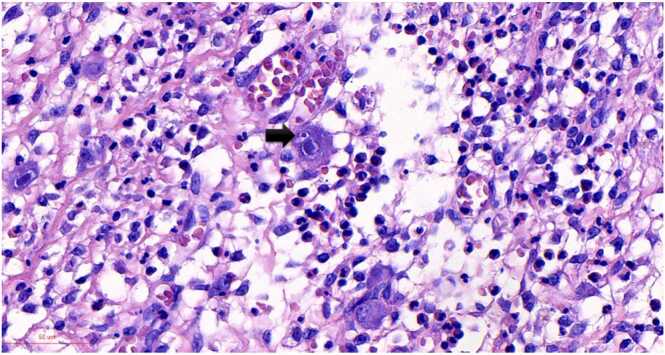
Intracytoplasmic inclusions of CMV infected cells. Note an intracytoplasmic inclusion in a CMV infected cell (arrow).

**Fig. 8:**
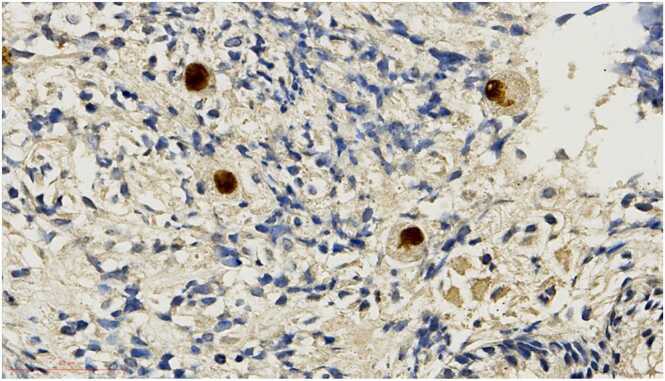
CMV immunostain. Immunohistochemical staining can be used to highlight CMV infected cells.

CMV colitis is the presence of CMV in the colon, causing inflammation and tissue damage. Following infection, disease results from reactivation of the latent virus, with CMV colitis being end-organ involvement of the colon [1]. CMV can lead to colitis in both immunocompetent and immunocompromised patients. Infection and reactivation are usually asymptomatic and self-limiting in immunocompetent patients but is a major cause of morbidity and mortality in immunocompromised patients due to reactivation in various organs [2]. In patients with acquired immunodeficiency syndrome (AIDS), the virus favors the colonic mucosa, resulting in CMV colitis being a common presentation of CMV reactivation in AIDS patients [2], [3].

CMV colitis is difficult to differentiate, as clinical presentation of CMV colitis mimics inflammatory bowel disease (IBD). The risk of misdiagnosis and subsequent treatment for IBD is detrimental to patients with CMV colitis. Additionally, symptoms of CMV colitis are non-specific, and there is a lack of the typical symptoms of CMV infection [3]. The gold standard for diagnosing CMV colitis is histological evaluation, including hematoxylin-eosin staining to visualize basophilic intranuclear inclusions bodies (“Cowdry bodies”) appearing as an “owl’s eye” specific to CMV or immunohistochemistry to identify CMV antibodies in infected cells [3], [4].

## Declaration of Competing Interest

The authors report no conflicts of interest.
